# An attempt to model the causal structure behind white matter aging and cognitive decline

**DOI:** 10.1038/s41598-023-37925-0

**Published:** 2023-07-05

**Authors:** Jan Willem Koten, Karl Koschutnig, Guilherme Wood

**Affiliations:** 1 Brain Imaging Facility of the Interdisciplinary Centre for Clinical Research of the University Hospital Rheinisch-Westfälische Technische Hochschule (RWTH), 52074 Aachen, Germany; 2 Department of Psychology, Karl-Franzens-University of Graz, 8010 Graz, Austria; 3 Biotechmed Graz, 8010 Graz, Austria; 4 COLIBRI Graz, 8010 Graz, Austria

**Keywords:** Nervous system, Cognitive ageing, Computational neuroscience, Neural ageing

## Abstract

In this diffusion tension imaging study, voxel wise structural equation modeling was used to unravel the relation between white matter, cognition, and age. Four neurocognitive ageing models describing the interplay between age, white matter integrity, and cognition were investigated but only two models survived an Akaike information criterion-based model selection procedure. The independent factor model predicts that there is no relation between white matter integrity and cognition although both systems are affected by age. The cognitive mediation model predicts that the relation between age and white matter integrity is mediated through cognition. Roughly 60% of the observed voxels were in agreement with the independent factor model while 16% of the observed voxels were in agreement with the cognitive mediation model. Imaging results of the latter model suggest that the deterioration of fibers—that connect the two hemispheres with each other—is partly caused by an age-related decline in cognitive functioning.

## Introduction

**Figure 1:**
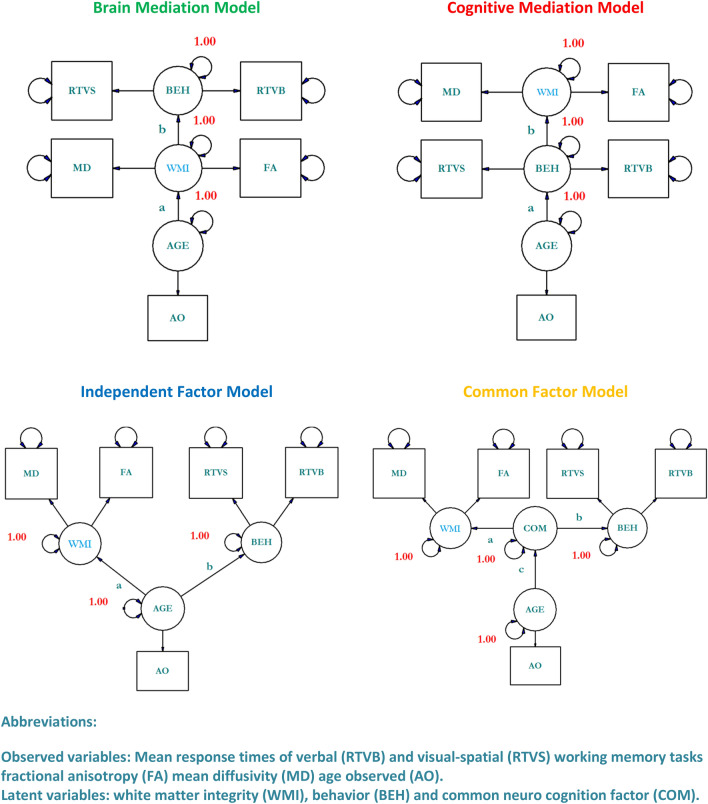
Four hypothetical brain aging mechanisms describing the relation between age, white matter integrity, and working memory. Note that FA values were multiplicated by − 1 to avoid anti-correlations.

Old age affects white matter integrity and cognition^1–12^. But it is unclear how age-related deteriorations in white matter integrity are related to age-related deteriorations in cognitive functions^12^. So far four hypothetical mechanisms have been proposed that predict how age, white matter, and cognition are related to each other (c.f. Fig. 1)^12^. The brain mediation model predicts that the relation between age and cognition is mediated by white matter. The cognitive mediation model predicts that the relation between age and white matter is mediated by cognition. The independent factor model predicts that there is no relation between white matter and cognition although both systems are affected by age. Finally, the common factor model predicts that the relation between age and white matter on the one hand and age and cognition on the other hand are mediated by a common neurocognitive factor. We have depicted the four models under study in Fig. 1.

We investigated these brain ageing hypotheses in a sample of 88 healthy individuals with an age between 18 and 89 years. Working memory measures and diffusion tension imaging data (DTI) were analyzed with a voxel-wise structural equation modeling (SEM) technique. A crucial element of an SEM analysis is the a priori specification of the model, which should be based on existing theory and evidence^13^. We followed this advice and strictly limited our analysis to models proposed by Bennett and Madden^12^.

Structural equation models estimate whether a hypothetical web of causal relations among multiple variables is consistent with the observed data. It is superior to conventional regression methods in the sense that it does not only focus on the existence of a single relation between two variables but also on the interplay between multiple variables in their entirety. It is possible to compare the statistical properties of distinct theoretical models—that have been estimated from the same data—and select the theoretical model that shows the highest agreement with the observed data.

The model distinguishes latent variables depicted as circles and observed variables depicted as squares. Latent variables are hypothetical constructs that are not directly observed. The potential existence of latent variables is indirectly infered from observed variables also known as indicators. The covariance structure between latent variables is estimated by means of observable variables and their intercorrelations. The structural equation model consists of two sub-models: the measurement model and the structural model. The measurement model describes the relations among the indicators and the latent variable in terms of covariance and is therefore closely related to confirmatory factor analysis. In the measurement model the relations among latent variables are less plagued by unsystematic measurement errors and other sources of nuisance that are specific to any one manifest measure^13^. The structural model—that is at the core of the method—describes the causal relations between endogenous and exogenous latent variables.

The chi-square model fit index reflects the statistical plausibility of the proposed SEM and is often associated with a p-value that is *not* significant when the hypothesized model is in agreement with the observed data. It is common practice to select the optimal SEM from a larger set of competing SEMs on the basis of Akaike’s information criterion (AIC). AIC is rooted in information theory and assumes that model representations are imperfect descriptions of reality. The discrepancy between the assumed model and the reality can be understood in terms of information loss. Models with a small degree of information loss are favored over models with a higher degree of information loss. The amount of information loss is understood as the trade-off between model complexity and goodness-of-fit. Complex models are penalized leading to solutions that are parsimonious. Among the set of models representing causal relationships the model associated with lowest AIC is considered the best approximation of reality.

To maximize content validity, one might argue that observations should measure complementary aspects of the latent variable. From a statistical point of view, one might argue that extremely low correlations indicate that the variables are too distinct in nature to be unified into one latent construct, while extremely high correlations might indicate that no information of interest is added to the model. Thus, ideally observed data should measure slightly distinct aspects of a phenomenon to cover the full breadth of their associated latent constructs.

## Results

### Preparatory steps

In this project, we defined the latent constructs behavior and white matter integrity (WMI). We used the response times of verbal (RTVB) and visual-spatial (RTVS) working memory tasks as observables for the latent construct behavior because these tasks are complementary in nature and correlated with age^14^. Moreover, a previous study showed that similar tasks exhibit high test-retest reliability and external validity when obtained during MRI scanning^15^. Statistical analysis of the RTVB and RTVS revealed that the average response times of both tasks showed differences—attesting the slightly distinct nature of the tasks—while the correlation among the two tasks was present attesting the complementary nature of both tasks (Supplementary Information 1). Furthermore, test–retest reliability of the two tasks was good to excellent confirming previous findings with similar tasks^15^. We considered to use fractional anisotropy (FA) and mean diffusivity (MD) as observables for the latent construct WMI. The latent construct WMI incorporates aspects of diffusion direction and diffusion rate which are believed to be related to white matter integrity^7^. FA reflects the fraction of the tensor that can be attributed to anisotropic (directional) diffusion^7^. Within this context, higher values usually indicate an increased directionality of diffusion, independently of diffusion rate. MD is the mean of all three axes of the diffusion ellipsoid which reflects the rate of water diffusion, independently of the directionality^7^. An important requirement for SEM is that the correlations between observed variables which load on to the same latent factor should share enough variance to produce interpretable results. The latter should be demonstrated for all voxels under study since correlations approaching zero may potentially corrupt model fit voxel-wise. We correlated FA and MD measures voxel-wise, that is, we correlated the 88 FA values per voxel with the 88 MD values per voxel. The FA measures were multiplied by − 1 to avoid anti-correlations between FA and MD. The histogram of the voxel-wise correlations between FA and MD is given in Supplementary Information 2. The median correlation of ~ 0.55 suggests that FA and MD indeed measure slightly different aspects of a similar phenomenon. Moreover, the correlation between FA and MD was indeed positive in almost all voxels suggesting that the interpretation of the latent construct WMI is similar irrespective of the voxel under study.

### Voxel-wise correlations between age, white matter, and cognition

**Figure 2:**
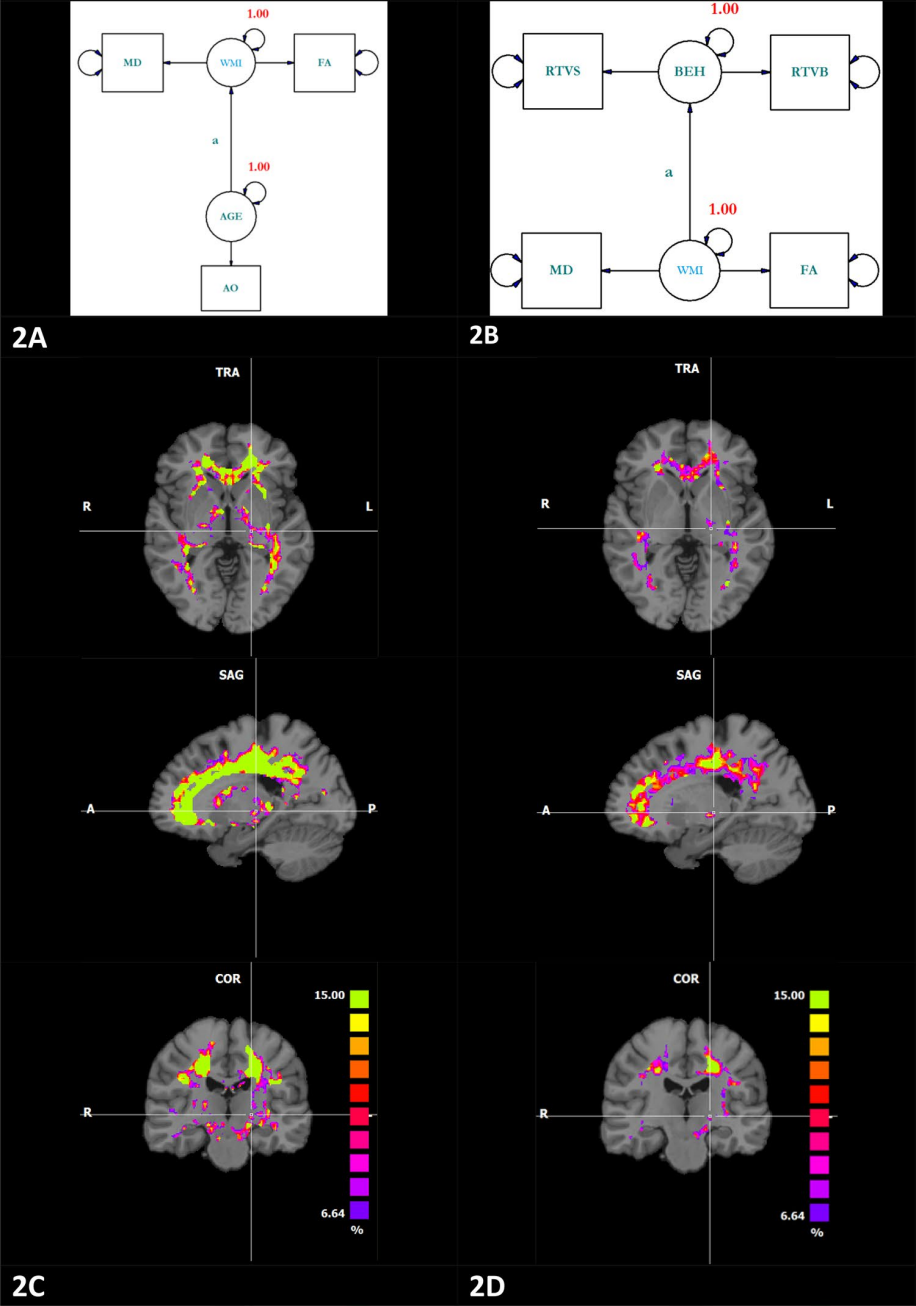
Structural equation models that conceptualize the relation between age and white matter integrity (**A**) or the relation between cognition and white matter (**B**). Note that FA was multiplicated by − 1. The statistical significance of the path “a” as depicted in (**A**) and (**B**) was tested and expressed as a chi-square difference statistic. The purple color (Chi-square difference = 6.64) refers to a p-value of 0.01 the green color (Chi-square difference = 15) refers to a p-value of 0.0001.

As a preparatory step, we assessed if response times are either correlated with age or white matter. We depicted the two SEMs in Fig. 2. For this analysis, we skeletonized the white matter, meaning that we only accepted voxels when all individuals showed a FA > 0.2. As mentioned above, FA values were multiplicated by − 1 meaning that a positive correlation between age and FA should be interpreted as an increase of diffusivity with age. Fit statistics for very simple SEM’s can be very high due to the saturated character of simple SEM’s. The use of fit statistics within this context is not advised^13^. Hence, we only tested the latent paths “a” as depicted in Fig. 2A,B respectively for their statistical significance^16^. The chi-square difference statistic with one degree of freedom is visualized in Fig. 2C,D. Effects of age on white matter and effects of WMI on cognition have been reported by many others. The effects of age on the brain are visible in almost the entire skeletonized white matter mask while the effects of cognition on WMI are mainly visible in the white matter tract along the dorsal route. But the nature of the statistically significant relations between cognition and WMI is not entirely understood since age is related to cognitive behavior as well. We tried to shed light on the true nature of the latter relation by means of SEM.

### Voxel-wise structural equation model comparisons

**Figure 3:**
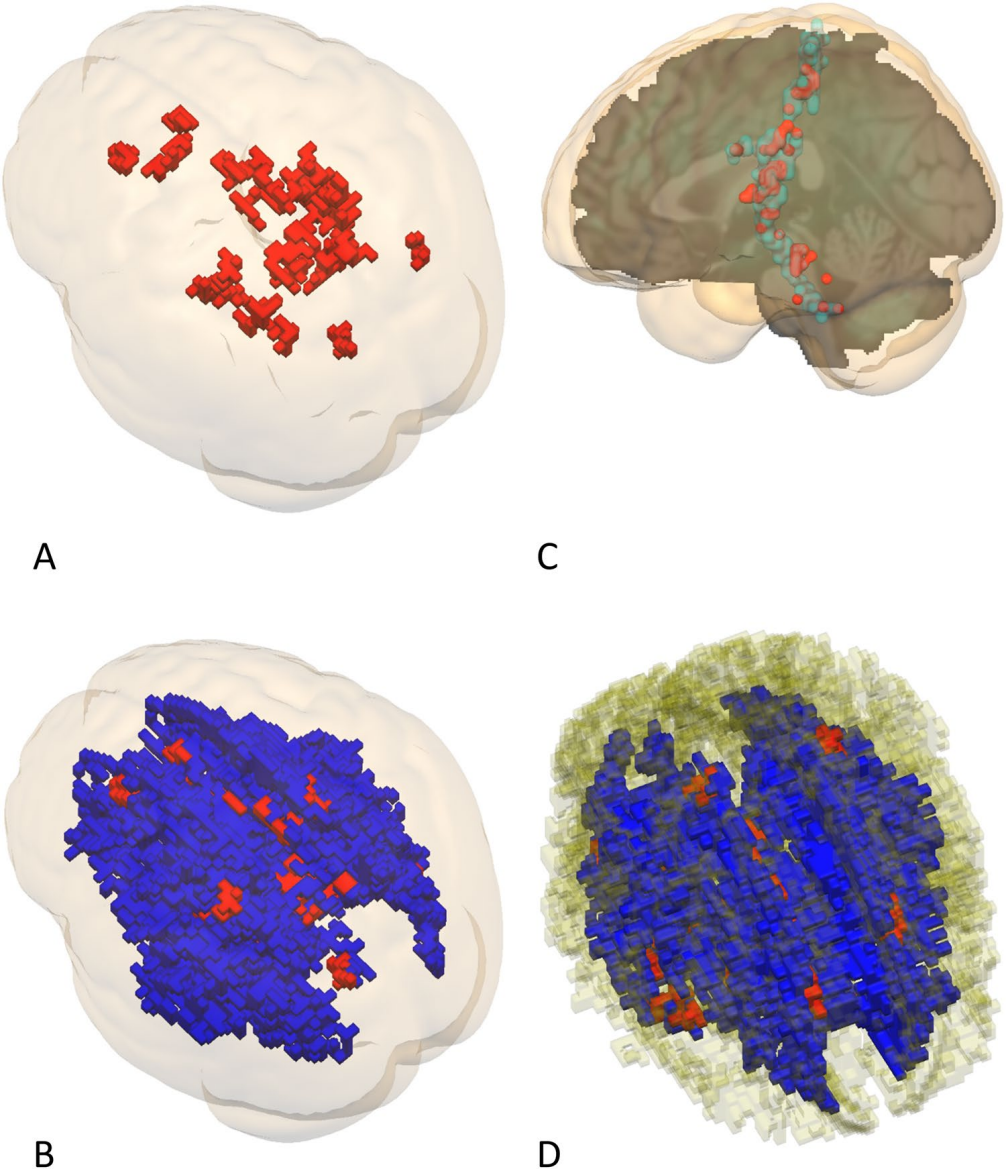
This figure depicts voxels that were selected by comparing AIC statics of the 4 ageing models under study. Only voxels with sufficient model fit (Chi-square p > 0.05) exhibiting non-corrupt correlations between latent factors (r < 1 and r > − 1) were depicted within the confines of the skeletonized white matter (FA > 0.2). Voxels related to the cognitive mediation model are depicted in red while voxels of independent factor model are depicted in blue. (**A**) Depicts the caudal dorsal bird's-eye view of voxels related to the cognitive mediation model thresholded at a volume of 300 mm^3^. (**B**) Depicts the caudal dorsal bird's-eye view of voxels related to the cognitive mediation model and independent factor model thresholded at a volume of 300 mm^3^. (**C**) Depicts voxels related to the cognitive mediation model that were found within the confines of the postcentral /cerebellum white matter tract as described by the IIT Human Brain Atlas thresholded at a volume of 120 mm^3^. (**D**) Depicts the rostral dorsal bird's-eye view of both models within the confines of the cingular fibers as described by the IIT Human Brain Atlas thresholded at a volume 300 mm^3^.

We inspected the fit statistics of the four brain maps that were related to the four neurocognitive models under study before we proceeded to the actual model comparisons. Histograms depicted in Supporting Fig. 2B show many voxels in the tails of the distributions with negative AIC values that exhibit abnormal distribution behavior. The latter was in particular true for voxels related to the “brain mediation” and the “common factor” maps. We removed voxels from the SEM map when model parameter estimates of the respective voxels were suspect (Supplementary Information 2). Voxels were deemed to be suspect when correlations between the latent factors were outside the − 1 to 1 range and when the Mx output reported ill conditioning. Application of this filter resulted in the removal of voxels that were mainly found in the left tails of the distributions (Supplementary Information 2). Moreover, all voxels of the “common factor map” were removed by the filter while AIC statistics of “brain mediation voxels” that remained after filtering suggested very poor model fit. The latter was formally investigated by thresholding the Chi-square associated model fit statistic at p > 0.05. As expected, brain mediation and common factor model maps were empty while the remaining cognitive mediation and independent factor maps covered vast portions of the white matter. Visualizations of the cognitive mediation and independent factor model fit maps (Chi-square associated model fit statistic p > 0.05) show that most voxels related to the cognitive mediation model were found within the confines of the independent factor model suggesting high spatial overlap between the maps in question (Supplementary Information 2). We selected the most appropriate brain aging model through a voxel wise AIC comparison of the four models under study within the white matter mask (FA > 0.2) and visualized the voxel of the selected model when it showed sufficient Chi-square related model fit p > 0.05 and correlations between the latent factors ranged between 1 and − 1 (Fig. 3). Not surprisingly, voxels described by the brain mediation or common factor model were absent. Sixteen percent of the skeletonized white matter voxels were in agreement with the cognitive mediation model while 60% of the skeletonized white matter voxels were in agreement with the independent factor model. The cognitive mediation model detected commissural fibers in the genu and splenium of the corpus callosum. In addition, fibers in the superficial white matter were detected that bordered to grey matter regions that are believed to be essential for working memory including parts of the superior parietal and superior frontal lobules (Fig. 3A,B). A conjunction analysis that estimated the common number of voxels detected by the cognitive mediation model and white matter tracts as present in the IIT atlas point to the relative importance of diverse tracts that all terminate in the hippocampus formation (Supplementary Information 5)^17^. Furthermore, we isolated voxels with sufficient fit that were part of the post central/cerebellar tract. Whether the relation between the post-central sulcus and the cerebellum truly exists is debatable due to the fragmentary character of the overall image (Fig. 3C). Finally, all the correlations between the latent construct WMI and working memory were positive.

The independent factor model detected voxels with sufficient model fit in most voxels of the skeletonized white matter mask (Fig. 3B). The top 50 structures are listed in Supplementary Information 6. Only a very small number of voxels detected with the cognitive mediation model located in the medulla exhibited a negative relation between age and white matter (Supplementary Information 3). Further analysis revealed that both the cognitive mediation model and the independent factor model showed high overlap with cingular fibers as defined in the IIT atlas (Fig. 3D)^17^.

### Post Hoc analysis results

As pointed out in the introduction, we tested SEM models that were based on a priori theories that were founded on the notion that narrow sense working memory is robustly correlated with ageing. Critics of this approach may object that two observed variables are possibly not enough to obtain sufficiently robust SEM results. Hence, we included a Number Stroop task—that was used as a distractor task during the working memory task—as a third measure of response behavior. This approach led to the broader latent construct “working memory + Stroop”. Just like the previous analysis that was based on 2 variables only, the brain mediation and common factor model were rejected. The introduction of the third variable led to a substantially higher number of “cognitive mediation voxels” that survived the model comparison procedure. Structures that were detected in the right hemisphere by the 2-variable model were extended into the ipsilateral hemisphere when 3 variables were used (Supplementary Information 4). The latter included prominent structures such as the commissural fibers in the genu and splenium of the corpus callosum as well as fibers related to the left hippocampus formation. Furthermore, gaps in structures mainly found in the right hemisphere were complemented when a third variable was used. However, structures that are believed to be essential for working memory such as frontal and parietal systems were partly lost when a third variable was added to the model.

## Discussion

The current study is to the best of our knowledge one of the first attempts in which the ageing brain is investigated on the basis of voxel-wise SEM. So far, most SEM studies tested models that were limited to a smaller number of ROI’s. In most cases, rather complex models were employed that show little similarity with models investigated in this study^10,18,19^.

We restrict our discussion to the current study due to inherent incompatibility with other brain ageing studies. In Fig. 2 we demonstrated that statistically significant relations between age and white matter as well as between response time and white matter exist, suggesting that increases in response times and age go along with deteriorations in white matter integrity. How can we interpret these relations within the context of brain aging models? The main strength of our study lays not in the detection of models but rather in their rejection.

We rejected the common factor model and brain mediation model. Mainly because we could find no voxels for which one of the models mentioned above could overrule all other models. This is rather remarkable since more than a quarter of a million voxels were investigated.

In principle, the independent factor model and the cognitive mediation model might qualify as valid descriptions of the aging brain. It is quite possible that in some cases models were wrongly ranked due to the inherent noise levels of the measures under study. However, one cannot exclude the possibility that the cognitive mediation model is correct in some cases. The frontal parietal system detected by the cognitive mediation model in the superficial white matter is known to be related to working memory which was the cognitive system under study. Furthermore, imaging results suggest that the deterioration of fibers—that connect the two hemispheres with each other—is partly caused by an age-related decline in cognitive functioning. Nonetheless brain ageing was in most voxels in agreement with the independent factor model. The independent factor model predicts that there is no relation between white matter and cognition although both systems are affected by age. This model can serve as the null hypothesis because the relation between age and cognition on the one hand and age and white matter on the other hand is very well-known. Recently it has been shown that relations between cognition and measures of the brain are at best very weak. Furthermore, brain wide associations studies might require substantial samples to obtain reproducible effects^20^. Currently, brain aging studies emerge that use very sophisticated (multi-modal) methods in larger samples. In principle, these studies are very suitable for SEM because SEM is able to integrate the distinct modalities into one model. Alternatively, sophisticated methods can be fused with SEM to provide a better insight into the mechanisms that lead to cognitive decline^21–26^. Given the large samples of these studies multimodal voxel-wise SEM is feasible.

Our study showed that the stability of voxel-wise SEM improves when the number of observed variables pointing to a specific latent variable is raised. This meant that the precise construct “working memory” was changed in the somewhat vague construct “working memory + Stroop”. It is not impossible that the increase of stability through a larger number of observed variables per latent variable might outweigh the potential loss of theoretical sharpness that goes along with it.

Our study shows several limitations. Our sample (n = 88) is only of medium size. Furthermore, the latent variable “common factor” of the common factor model was only defined in an abstract way. The common factor model may in potential fit with the MRI data when the latent construct “common factor” is operationalized with variables such as genes, grey matter integrity, etc. It was not within the reach of this study to add such variables to the model. In this study, we used FA and MD as observed variables for the latent construct WMI. This can be criticized because both measures are based on the three eigenfactors lambda 1–3 and therefore not independent. We nonetheless think that the unification of both measures leads to a meaningful latent construct because the latter encapsulates diffusion direction and diffusion rate. As an alternative, one might consider a riskier approach that includes axial diffusivity which is based on lambda 1 and radial diffusivity which is based on lambda 2–3. Such an approach can be thorny within the context of SEM because radial diffusivity can be positively and negatively correlated with axial diffusivity meaning that a larger set of voxels is inherently impossible to model^7^. The risky approach is attractive despite its potential problems but it was not within the scope of this pioneering study to investigate it. We conclude that future DTI studies of the ageing brain may focus on cognitive mediation or independent factor models that are preferably operationalized with a larger number of behavioral measures.

## Methods

This study is a reanalysis of a previous published DTI study^27^. All the descriptions of the sample composition, sample properties and scanner parameters of the study mentioned above apply to this study as well.

### Participants

Prior to the scanning procedure a health-screening questionnaire was administered. None of the participants reported a history of chronic illnesses or heart and brain surgery. A scanner compatible lens system was used when individuals used lenses in daily life. None of the participants showed signs of dementia or depression as attested by the German version of the Mini Mental State Examination and the Depression-scale (Allgemeine Depressionsskala)^28,29^ . All participants received monetary reimbursement at the end of the testing. The initial sample was stratified by age, sex, and education and reflected the distribution properties of the Austrian population. The sample included 88 healthy adults with an age range between 18 and 89 (M = 45.47, SD = 18.37, women = 54) The sample consisted of 3 age groups (Table 1). All but 10 participants were right-handed, out of these 10 three use their right hand to write.

**Table 1:** This table gives the demographic description of the sample in use.

Age interval (years)	Mean age ± SD (years)	Number of participants (males/females)	Education (< 12 years/ > 12 years)
18–39	26.69 ± 5.34	36 (17/19)	22/14
40–59	49.04 ± 6.31	27(8/19)	16/11
60–89	68.64 ± 5.96	25(9/16)	13/12

### Task

Several variants of filled delay tasks also known as Peterson tasks were performed^14^. In this study, participants were confronted with spatial and verbal filled delay tasks. Response behavior on these tasks is believed to be correlated with age^14^. Prior to scanning, participants were trained until they performed confidently. This procedure guarantees that reaction times and not training effects were measured.

**Figure 4:**
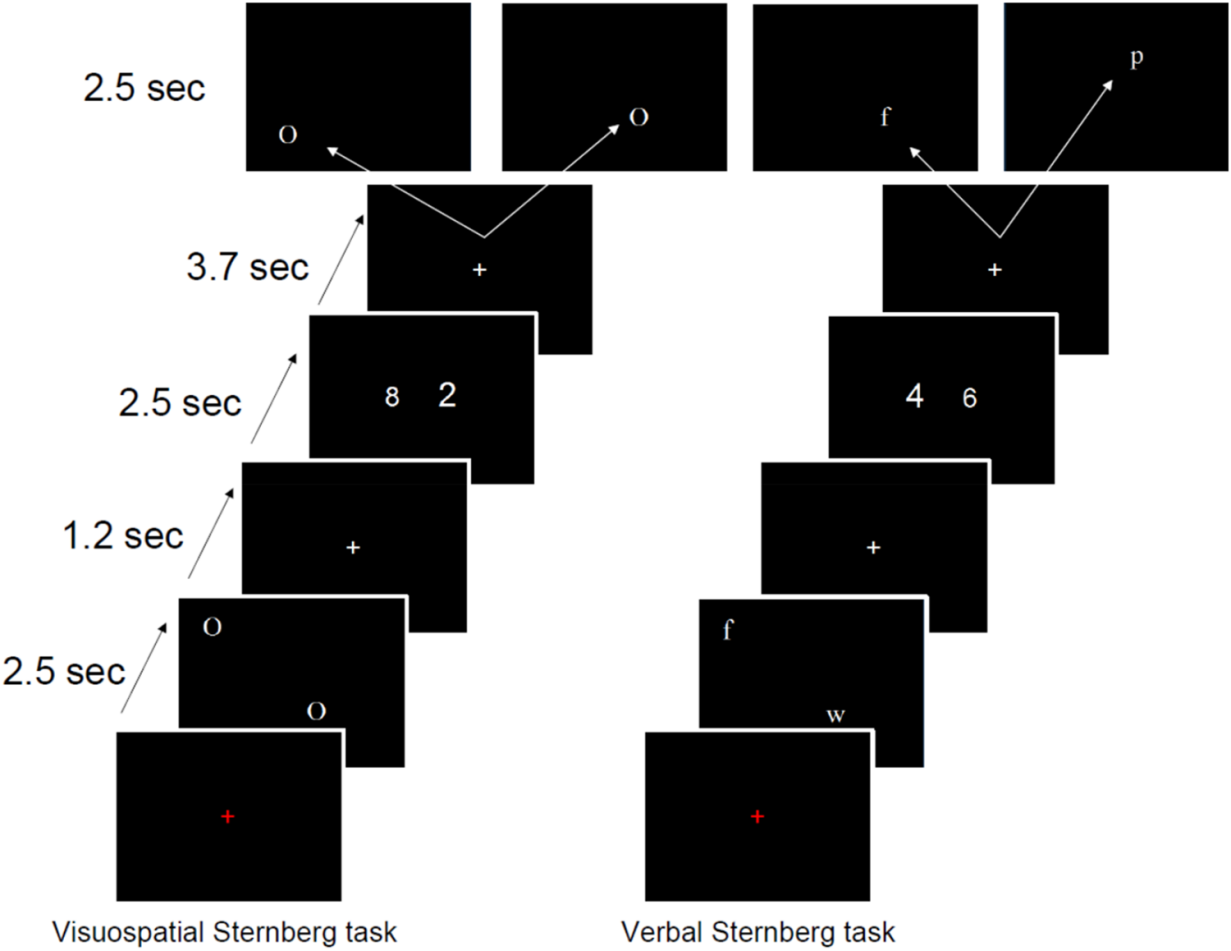
This figure depicts the visual spatial (left) and verbal (right) filled delay experiments used in this study. The last slides of the task cycle visualize two possible slides. For the spatial experiment, the left slide is incorrect while the right slide is correct while the opposite is true for the verbal task.

In short: individuals had to retain two letters or two spatial positions in working memory while performing a number stroop task. During the encoding phase, a set of two letters or two spatial positions was memorized; subsequently, individuals had to perform a number Stroop task; finally, individuals had to press a button with the right index finger when a newly presented item was part of the previously presented item set. The left finger was used in case of non-correspondence. The number Stroop tasks consisted of two simultaneously presented Arabic numbers that were presented in two physical sizes. Individuals had to identify the larger numerosity and use the right finger when the larger number was found on the right side of the screen and vice versa. In the congruent condition, the larger numerosity corresponded with the larger physical size of the Arabic digit while the opposite was true for the Stroop condition. All phases were separated by fixation crosses. We visualized the exact course of the behavioral experiment in Fig. 4.

The verbal and spatial items presented during the encoding phase were spatially randomized and positioned within the cells of an invisible 4 * 4 matrix. The relevant spatial positions of the spatial working memory task were indicated with the symbol “O”. It is well established that eye movements are correlated with fontal parietal brain activations. Hence, the letters used for the verbal memory task and the “O” used for the spatial memory task were presented at the same positions to control for eye movements. It should be mentioned that the spatial position of the letter was irrelevant for the verbal task.

Every task cycle was presented 24 times in two separated fMRI sessions that were interrupted by a pause outside of the scanner leading to 2*24 = 48 reproductions for every task cycle. The 24 responses of the first and second scanning sessions were averaged and entered into an intraclass correlation model to estimate the test–retest reliability of the task. Next, the 48 responses per task were averaged and used for further analysis.

### Scanner parameters and FSL preprocessing

All data were acquired on a 3T Siemens Skyra MRI scanner with a 32-channel head coil. For diffusion weighted imaging a single-shot echo planar imaging sequence was used. The sequence parameters were: TR/TE/flip angle = 6600 ms/95 ms/90°, matrix size = 122 × 122 mm, FoV = 240 mm, 50 transverse slices of 2 mm thickness were measured, slice gap = 0.5 mm, *GRAPPA* acceleration factor = 2. 64 diffusion sensitizing gradient directions were applied (b value = 1000 s/mm^2^) and one non-diffusion weighted image (b value = 0 s/mm^2^). The total acquisition time was 7 min 30 s. Data were converted from Dicom to NifTI format. Moreover, text files were created that contained vector and b value information. The resulting images were preprocessed using the FSL software in a standard multi-step procedure: (a) motion and eddy current correction (b) removal of the skull and non-brain tissue using the Brain Extraction Tool (c) voxel-wise calculation of diffusion tensors and computation of FA and MD maps using DTI fit^30^. Each participant's FA volume was brought into a 1 × 1 × 1 mm^3^ common space (Montreal Neurological Institute space; MNI152) via the FMRIB58_FA template using nonlinear registration tool (FNIRT). The same voxelwise statistical analysis was carried out for mean diffusivity (MD) by applying the nonlinear warps obtained from FA images.

### Structural equation modeling

The structural equation models were estimated in Mx. In total, four models were depicted and compared. Every model was tested in two variants. For the first variant, the latent construct “working memory” was constructed from the observed variables spatial working memory and verbal working memory. For the second variant, the latent construct “working memory + Stroop” was constructed from the observed variables spatial working memory, verbal working memory and a number Stroop task. The latent construct working memory and cognition are abbreviated as BEH in the graphs depicted in Fig. 2. Since two observations of the Stroop task existed that were both tested two times, we decided to average the 2*48 responses per subject. The latter has the advantage that verbal, spatial and executive functions contribute equally to the latent factor cognition.

### SEM DTI pipeline

The pipeline consisted of several routines that link FSL output with MATLAB, Mx, and BVQX^16,30–32^. This method was established in a previous study that was executed in mesh space^33^. We adapted this method such that voxelwise SEM could run on a small high-performance cluster consisting of 96 processors. We only imported a voxel to our pipeline when FA > 0.2 (strict cut off) in all individuals under study. FA and MD data show anti-correlated behavior. Hence FA data were multiplied by minus one, meaning that a positive correlation between age and FA should be interpreted as an increase of diffusivity with age. Next, DTI, RT, and age data were scaled from zero to ten. This is an important step because the starting values for Mx should be given within a predictable data range. From the scaled data covariance matrices were computed which is the standard input format for Mx^16^. The relevant Mx and MATLAB scripts are given in supplementary information. Mark that the brain mediation model, cognitive mediation model and independent factor model only differ with respect to the A matrix which is depicted in red. In some cases, Mx optimization procedures fail. Fortunately, the Mx output delivers three classes of warnings that indicate ill conditioning. IFAIL = 1 (code GREEN), most likely, the correct solution. IFAIL = 4 (code BLUE—I ran out of breath), this warning indicates that a larger number of iterations is needed for the minimization procedure. IFAIL = 3 (code RED—bad news) occurs in connection with constraints, normally they have been mis specified so that they are impossible to satisfy. In our analysis, voxels that were plagued by warnings were replaced by zero and therefore removed from the resulting image matrix. We inspected the images for the direction of the path covariance’s (positive or negative). The depicted images exhibited positive relations in almost all cases. In this analysis, we mainly focused on Chi-square model statistics because they are of vital importance in SEM. The Chi-square model fit statistic reflects the statistical plausibility of the proposed model. The Chi-square model fit is often expressed as a p-value that is not significant when the proposed model is sound. It is common practice to select the optimal model from competing models on the basis of Akaike’s information criterion (AIC). AIC gives the compromise between model complexity and model fit. Thus, simple models tend to be favored over complex models when similar Chi-square statistics exist. The paths between variables can be tested for their statistical significance through model fit comparison. Within this context, the model fit of a model with and without the path of interest is directly compared. The loss of model fit informs about the statistical significance of a specific path. All the images in this study were thresholded at 300 mm^3^. This appeared to be a rather conservative policy in particular when the four models under study were compared together on the basis of AIC. The resulting maps were exported from MATLAB to BVQX for visualization purposes.

### Overlap statistic

We estimated the percentage of the skeletonized white matter (FA > 0.2) that was detected by a specific model after AIC comparison given that the model fit yielded a non-significant result (Chi-square p > 0.05) and the correlations among the latent variables were not smaller than − 1 and greater than 1.

$${\text{Overlap }} = \left( {{\text{number of detected voxels/number of voxels with FA }} > 0.{2}} \right) \, *{ 1}00$$

### Ethics approval

All individuals provided written informed consent. The research was executed according to the guidelines of the Declaration of Helsinki. The study was approved by the Ethics committee of the University of Graz under GZ 39/31/63 ex 2011/12.

## Supplementary Information

Supplementary Information.

## Data Availability

The datasets generated and/or analyzed in the current study are available from the corresponding author upon request.

## Code availability

We would like to state that we used the standalone version of Mx instead of open Mx as implemented in R. We provide the Mx and MATLAB code used in this project in the supplementary information.
