# Supplementary material for: An attempt to model the causal structure behind white matter aging and cognitive decline
Source: Sci Rep. 2023 Jul 5;13:10883. doi: 10.1038/s41598-023-37925-0 (PMC10322973; doi:10.1038/s41598-023-37925-0)
Supplement: Supplementary file 1 — Supplementary Information. [file 41598_2023_37925_MOESM1_ESM.docx]

**Supplementary Information**

**An attempt to model the causal structure behind white matter aging and cognitive decline.**

**Jan Willem Koten Jr ^1,2^ *, Karl Koschutnig ^2,3^, Guilherme Wood ^2,4^**

**Supplementary information 1**

The two-response time means of the test and retest phase of the experiment were used for a test-retest reliability analysis. Test-retest reliability of the tasks under study was good to excellent (ICC for spatial working memory task = 0.72; ICC for the verbal working memory = 0.76). Next, we averaged the data of the test-retest phase for further analysis including SEM. The paired t-test revealed substantial differences in response time between the verbal and the spatial working memory task (p<0.001) confirming that these tasks are indeed distinct in nature. But the correlation (r = 0.79) between the averaged response times of the spatial and verbal working memory tasks suggests that individual differences in response time of the tasks are driven by a common factor. One common factor might be age.


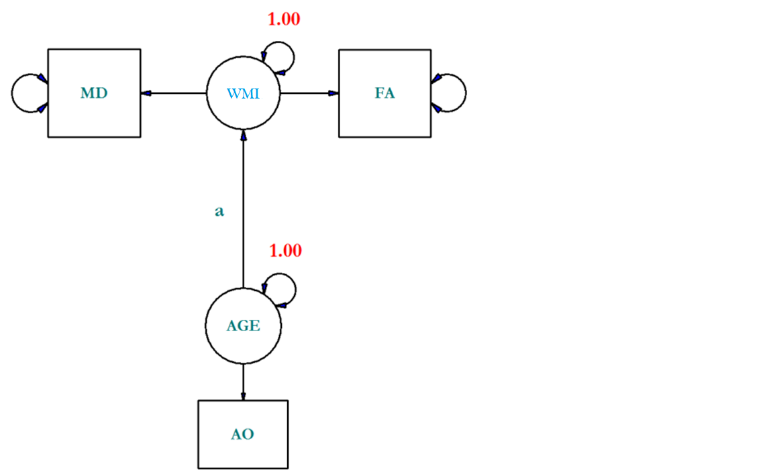


Supplementary Figure S1: depicts the relation between age and working memory

The latter was investigated by means of an SEM as depicted in supplementary figure 1 that describes the relation between age and the two reported measures of verbal and spatial working memory. This model fitted extremely well (p=1.0) and the path “a” between the latent factor age and the latent factor behavior was highly significant p<0.001. This suggests that a strong relation between age and measures of working memory exist.

**Supplementary Information 2**

| 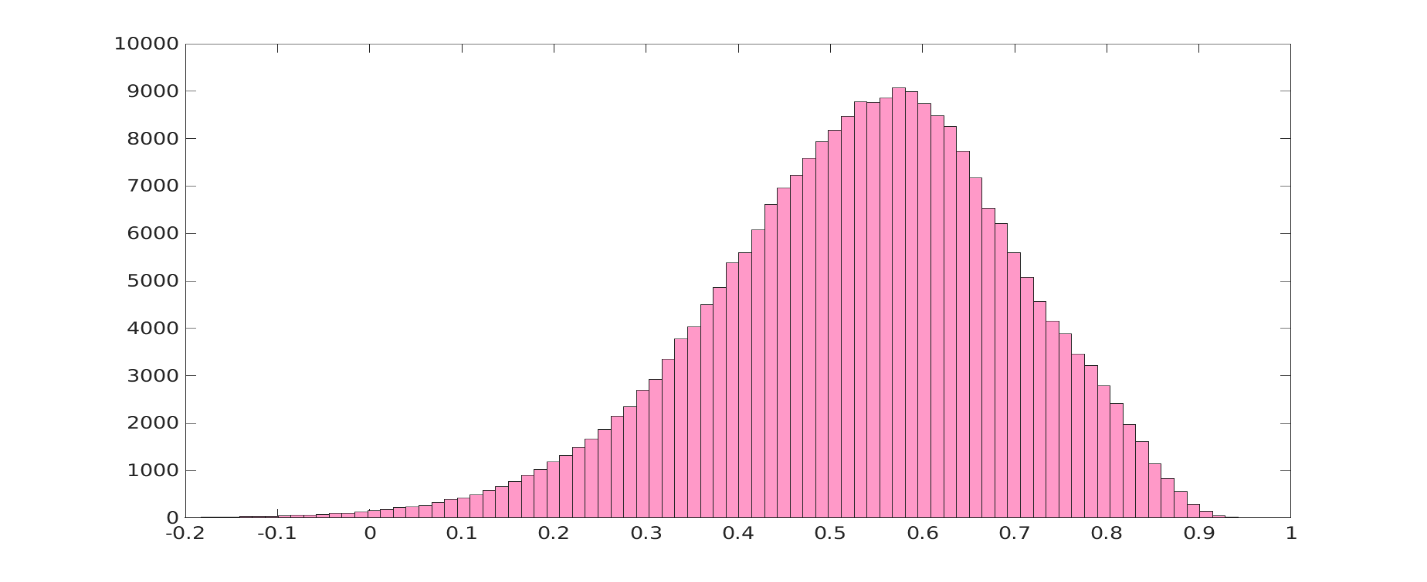 | |
| --- | --- |
| **A** | |
| 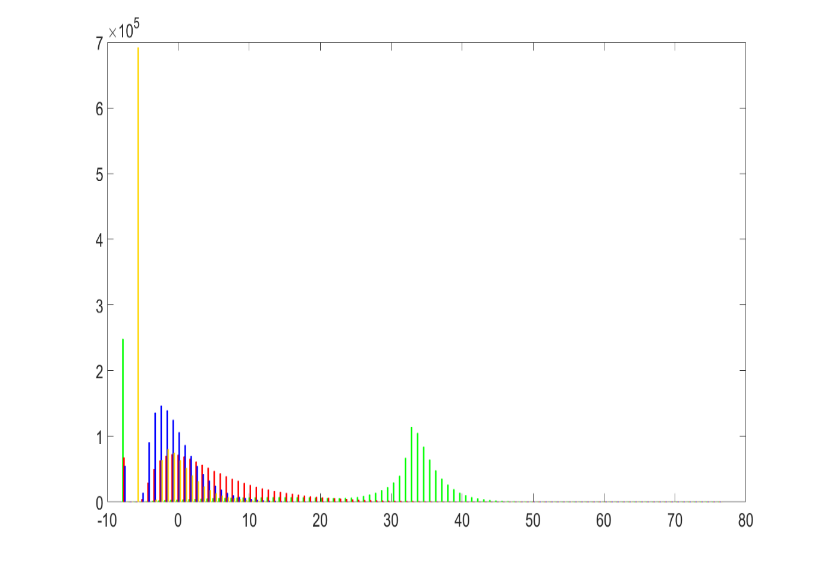 | 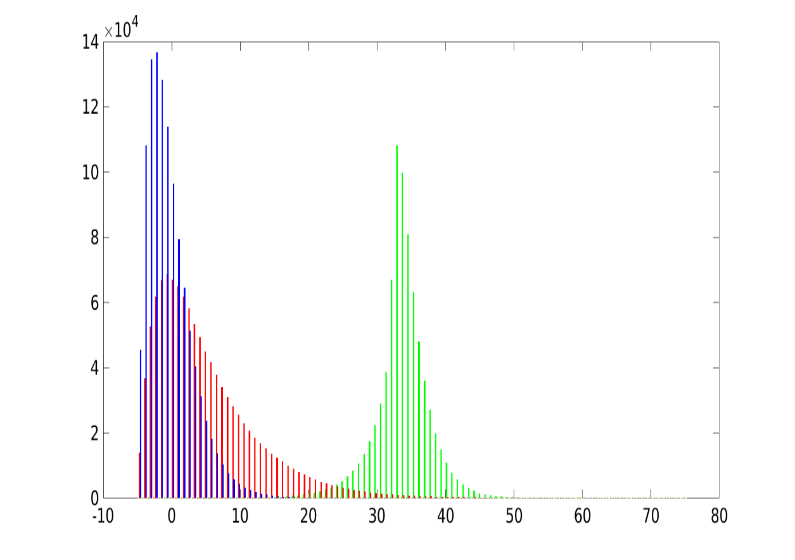 |
| **B** | **C** |
| 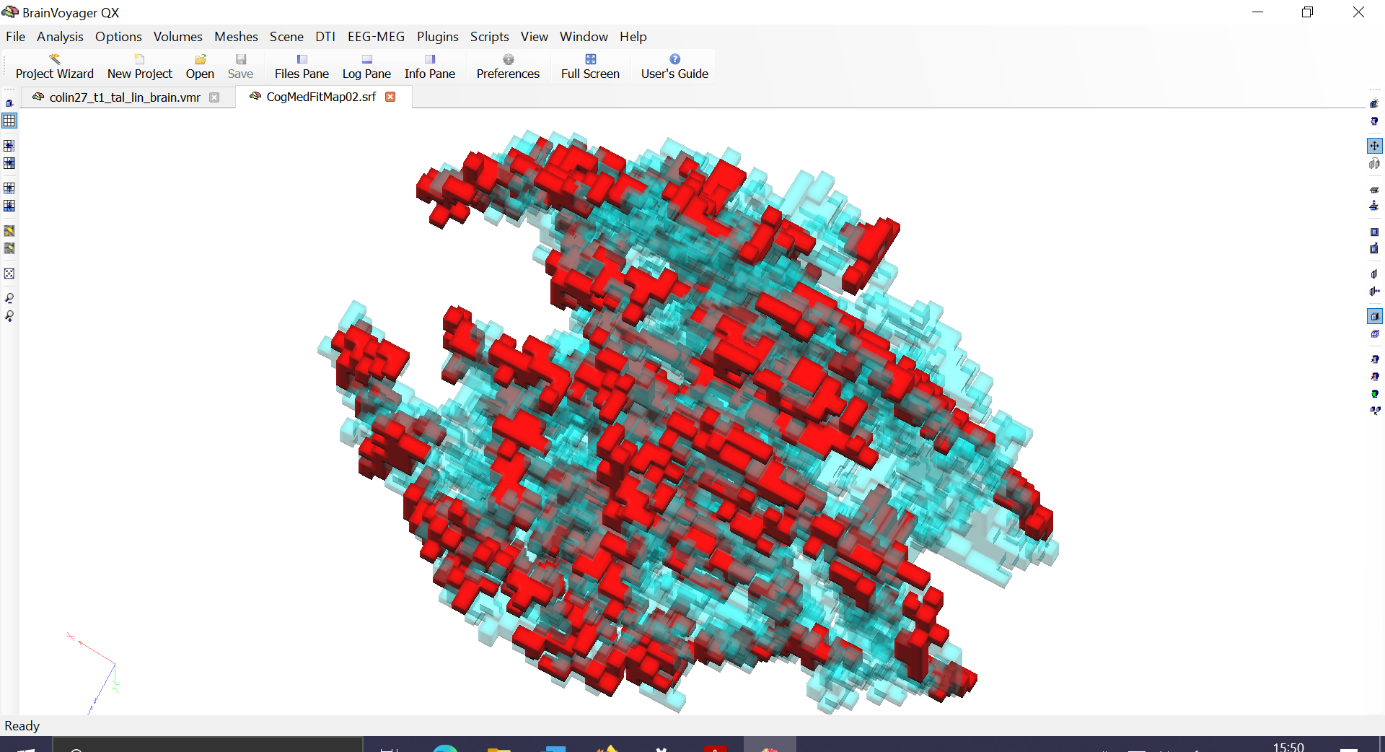 | |
| **D** | |

Supplementary Figure S2: reports key statistics that were used to judge whether the requirements of voxel wise structural equation modeling where fulfilled.

Panel A: Histograms of the voxel wise correlation between FA and MD. Note that FA values were multiplicated with -1 to ensure that both FA and MD correlate positive with the latent construct WMI. Three permille of the voxels showed anti correlated behavior which resulted inherently in poor model fit.

Panel B: Histograms of the voxel wise AIC statistics related to the Brain mediation model (green) cognitive mediation model (red) independent factor model (blue) common factor model (yellow). This histogram includes voxels that might exhibit ill conditioning.

Panel C: Histogram of the voxel wise AIC statistics related to the Brain meditation model (green) cognitive mediation model (red) independent factor model (blue) common factor model (yellow). This figure reports AIC voxels after filtering. We removed data from the SEM maps when the correlations between the latent factors were greater than one and smaller than minus one and when the mx output reported ill conditioning of the model.

Panel D: Rostral dorsal bird’s eye view depicting voxels with sufficient model fit (chi square p>0.05) related to cognitive mediation model (red) and independent factor model (transparent blue). We removed data from the SEM maps when the correlations between the latent factors were greater than 1 and smaller than -1 and when the mx output reported ill conditioning of the model.

The outlying behavior of the AIC distributions at the left side of the histograms as depicted in figure 2B suggest ill conditioning of the SEM. This is in particularly the case for the common factor model. The comfortable fit statistics of the independent factor model (blue) suggest that a common factor is not very likely. The latter is indeed reflected in the poor behavior of the common factor AIC distribution depicted in yellow. Mark that the distributions of distinct models exhibit normal behavior when suspect voxels are omitted from the maps (figure 2C). the sanctioning procedure led to a total collapse of the common factor model.

**Supplementary Information 3**

| 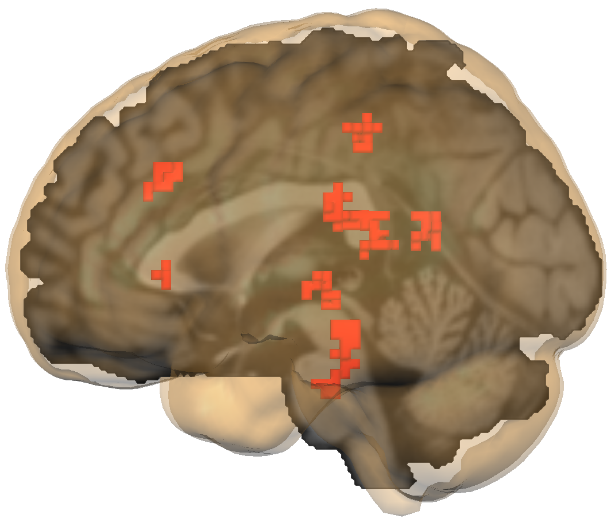 | 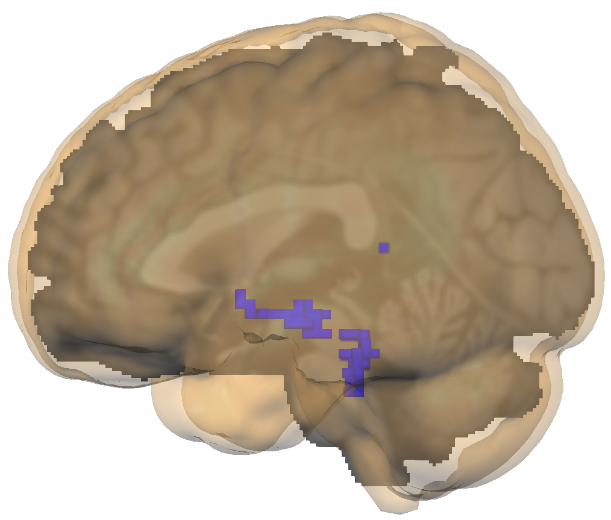 |
| --- | --- |
| **A** | **B** |

Supplementary Figure S3 depicts voxels detected with sufficient fit related to the cognitive mediation model (red) and independent factor model (blue).

Panel A: Depicts the lateral view of voxels related to the cognitive mediation model thresholded at a volume of 300 mm³. This image depicts voxels that exhibited a positive correlation between the latent factor and age.

Panel B: Depicts the lateral view of voxels related to the independent factor model thresholded at a volume of 300 mm³. This image depicts voxels that exhibited a negative correlation between the latent factor and age.

**Supplementary Information 4**

| 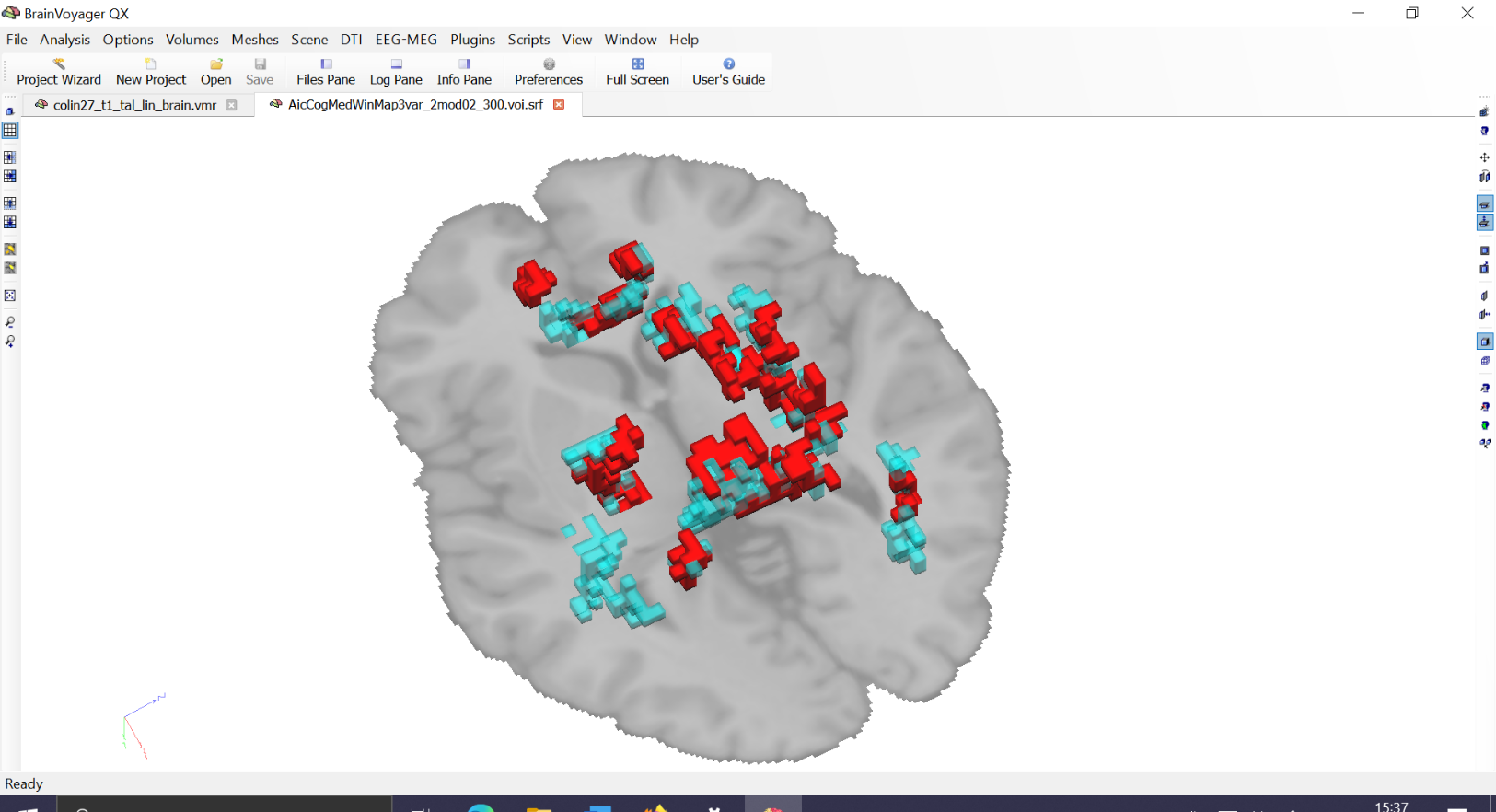 | 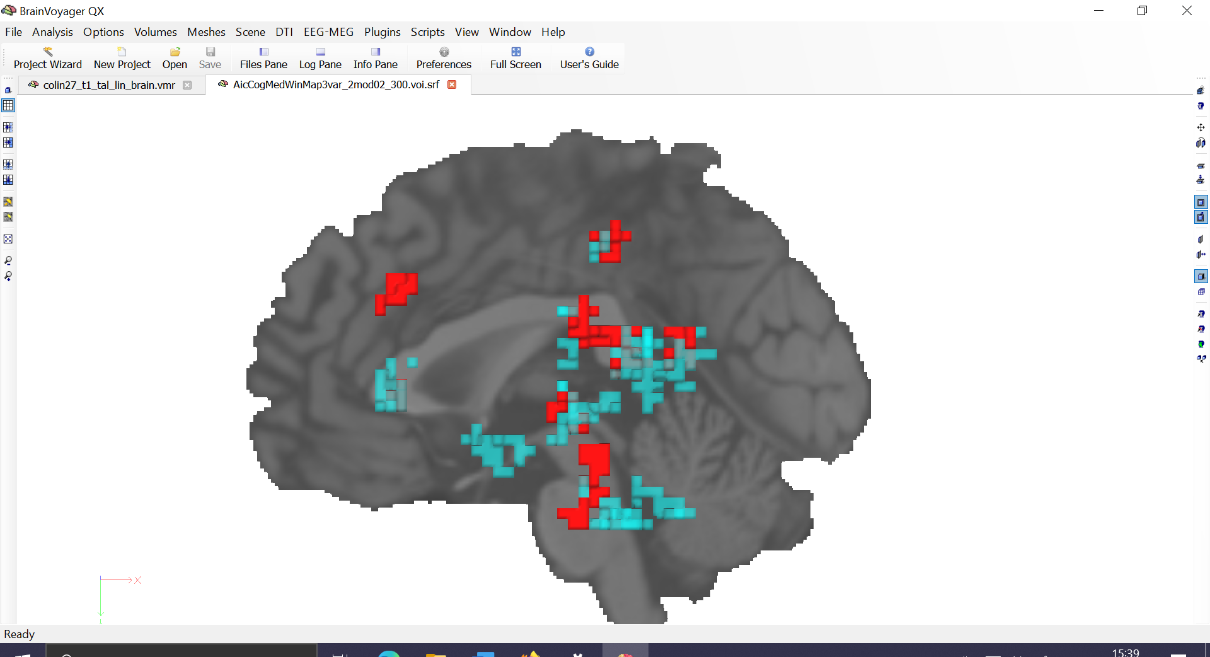 |
| --- | --- |
|  | **B** |
|  | 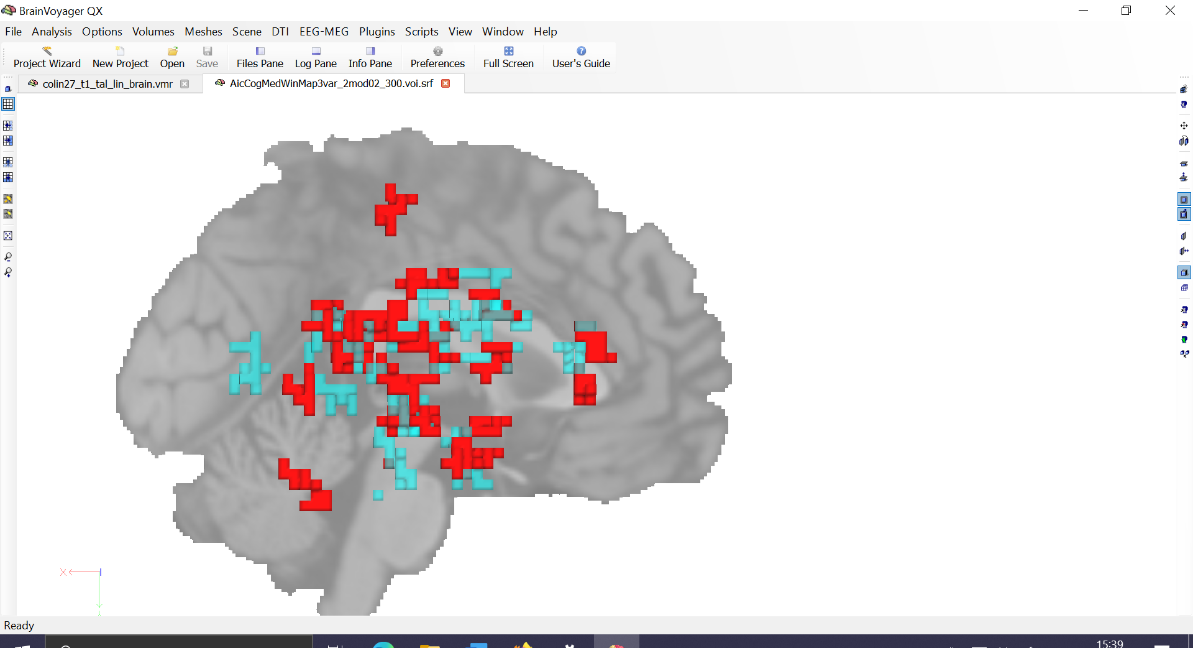 |
| **A** | **C** |

Supplementary Figure S4 depicts voxels related to the cognitive mediation model that were selected by comparing AIC statistics of 4 ageing models. Only voxels with sufficient model fit (chi square p>0.05) and correlations among latent factors < 1 and > -1 detected within skeletonized white matter (FA>0.2) are depicted. Voxels related to the cognitive mediation model employing measures of verbal and spatial working memory are depicted in red while voxels employing two measures of working memory and an additionally distractive stoop task are depicted in transparent blue. All images were thresholded at a volume of 300 mm³

**Panel A: Caudal dorsal view**

**Panel B: Lateral left hemispheric view**

**Panel C: Lateral right hemispheric view**

**Supplementary** **Information 5**

| name | code | Percentage filled | number of voxels | mean model fit |
| --- | --- | --- | --- | --- |
| Right-Hippocampus_ctx-rh-insula | 4783 | 20 | 1704 | 0.221 |
| Right-Putamen_Right-Hippocampus | 4547 | 17 | 1823 | 0.208 |
| Right-Hippocampus_ctx-rh-lateralorbitofrontal | 4760 | 17 | 1048 | 0.214 |
| ctx-lh-caudalanteriorcingulate_Left-Hippocampus | 240 | 16 | 938 | 0.212 |
| Left-Pallidum_Left-Hippocampus | 3940 | 16 | 1145 | 0.233 |

Supplementary Table S5 describes the anatomic properties of the cognitive mediation map. Here we report the number of voxels that were within the confines of the IIT reference white matter atlas. Name and tract code are according to the nomenclature of the mentioned atlas as described in: (https://www5.iit.edu/~mri/Home.html). The number of voxels refers to the absolute number of voxels that were commonly present in the SEM and atlas maps, while percent filled describes the extent to which white matter atlas maps were filled with SEM voxels. Finally, we report the mean model fit of the voxels found within the confines of the atlas maps.

**Supplementary** **Information 6**

| name | code | Percentage filled | number of voxels | mean model fit |
| --- | --- | --- | --- | --- |
| ctx-lh-isthmuscingulate_ctx-lh-medialorbitofrontalroi | 913 | 59 | 1653 | 0.226 |
| ctx-lh-isthmuscingulate_Left-optic-tract | 987 | 56 | 1288 | 0.226 |
| ctx-lh-posteriorcingulate_Left-Cerebellum-Cortex | 2235 | 48 | 1436 | 0.210 |
| ctx-lh-posteriorcingulate_Axial-section-through-medulla | 2285 | 47 | 3951 | 0.215 |
| ctx-lh-parahippocampal_Left-Putamen | 1538 | 47 | 1167 | 0.224 |
| ctx-lh-caudalanteriorcingulate_ctx-rh-medialorbitofrontal | 262 | 46 | 2869 | 0.224 |
| Left-Caudate_ctx-rh-frontalpole | 3780 | 46 | 2943 | 0.228 |
| ctx-lh-caudalanteriorcingulate_Left-Hippocampus | 240 | 45 | 2603 | 0.223 |
| ctx-lh-parahippocampal_ctx-lh-paracentral | 1516 | 45 | 2928 | 0.220 |
| Left-Hippocampus_Left-optic-tract | 4087 | 45 | 892 | 0.239 |
| ctx-lh-caudalanteriorcingulate_ctx-lh-medialorbitofrontal | 213 | 44 | 2899 | 0.220 |
| Left-Putamen_Left-Hippocampus | 3840 | 44 | 4799 | 0.225 |
| ctx-lh-insula_Left-Hippocampus | 3440 | 43 | 4920 | 0.221 |
| Left-Caudate_Right-Thalamus-Proper | 3743 | 43 | 2757 | 0.206 |
| ctx-lh-caudalanteriorcingulate_Left-Accumbens-area | 242 | 43 | 1102 | 0.239 |
| ctx-lh-superiorparietal_Left-optic-tract | 2887 | 42 | 2085 | 0.216 |
| Left-Hippocampus_Left-Amygdala | 4041 | 42 | 2936 | 0.229 |
| Right-Pallidum_ctx-rh-parahippocampal | 4664 | 42 | 1060 | 0.201 |
| ctx-lh-frontalpole_ctx-rh-lateralorbitofrontal | 3160 | 42 | 2934 | 0.217 |
| Right-Pallidum_Right-Hippocampus | 4647 | 42 | 3001 | 0.213 |
| ctx-lh-isthmuscingulate_Axial-section-through-medulla | 985 | 42 | 5396 | 0.219 |
| ctx-lh-rostralanteriorcingulate_ctx-rh-medialorbitofrontal | 2562 | 42 | 4155 | 0.222 |
| Left-Pallidum_Left-Hippocampus | 3940 | 42 | 3049 | 0.214 |
| ctx-lh-medialorbitofrontal_ctx-rh-rostralmiddlefrontal | 1375 | 41 | 5976 | 0.209 |
| ctx-lh-rostralanteriorcingulate_ctx-rh-lateralorbitofrontal | 2560 | 41 | 2516 | 0.218 |
| ctx-lh-parsorbitalis_ctx-rh-lateralorbitofrontal | 1860 | 41 | 1457 | 0.226 |
| Left-Pallidum_Right-Pallidum | 3946 | 41 | 5754 | 0.219 |
| ctx-lh-superiorfrontal_ctx-rh-frontalpole | 2780 | 41 | 5687 | 0.218 |
| ctx-lh-frontalpole_ctx-rh-medialorbitofrontal | 3162 | 41 | 4602 | 0.219 |
| ctx-lh-rostralanteriorcingulate_ctx-lh-frontalpole | 2531 | 41 | 2097 | 0.203 |
| Right-Pallidum_Right-optic-tract | 4688 | 41 | 1845 | 0.213 |
| ctx-lh-superiorfrontal_ctx-rh-lateralorbitofrontal | 2760 | 41 | 6739 | 0.214 |
| Left-Thalamus-Proper_Left-Accumbens-area | 3642 | 41 | 1345 | 0.232 |
| ctx-lh-rostralanteriorcingulate_ctx-lh-insula | 2534 | 41 | 2606 | 0.231 |
| ctx-rh-parahippocampal_ctx-rh-precentral | 6472 | 41 | 3410 | 0.235 |
| ctx-lh-insula_Left-Amygdala | 3441 | 41 | 859 | 0.227 |
| Left-Pallidum_Left-optic-tract | 3987 | 41 | 2390 | 0.217 |
| Left-Pallidum_Left-Amygdala | 3941 | 40 | 2085 | 0.220 |
| ctx-lh-parahippocampal_Left-Thalamus-Proper | 1536 | 40 | 3114 | 0.210 |
| ctx-lh-isthmuscingulate_Left-Hippocampus | 940 | 40 | 6355 | 0.209 |
| Right-Pallidum_Right-Amygdala | 4648 | 40 | 2503 | 0.213 |
| Left-Putamen_ctx-rh-superiortemporal | 3878 | 40 | 1778 | 0.211 |
| Left-Pallidum_Right-Thalamus-Proper | 3943 | 40 | 6929 | 0.219 |
| Left-Caudate_Left-Pallidum | 3739 | 40 | 7450 | 0.207 |
| ctx-lh-paracentral_Left-Cerebellum-Cortex | 1635 | 40 | 5509 | 0.217 |
| ctx-lh-parahippocampal_Left-Pallidum | 1539 | 40 | 1800 | 0.213 |
| ctx-lh-superiorfrontal_ctx-rh-middletemporal | 2763 | 40 | 6674 | 0.208 |
| ctx-lh-superiorfrontal_Left-Cerebellum-Cortex | 2735 | 40 | 5912 | 0.216 |
| ctx-lh-lateralorbitofrontal_ctx-rh-frontalpole | 1180 | 40 | 3503 | 0.222 |
| ctx-rh-paracentral_Axial-section-through-medulla | 6585 | 40 | 9250 | 0.231 |

Supplementary Table S6 describes the anatomic properties of the independent component map. Here we report the number of voxels that were within the confines of the IIT reference white matter atlas. Name and tract code are according to the nomenclature of the mentioned atlas as described in: (https://www5.iit.edu/~mri/Home.html). The number of voxels refers to the absolute number of voxels that were commonly present in the SEM and atlas maps, while percent filled describes the extent to which white matter atlas maps were filled with SEM voxels. Finally, we report the mean model fit of the voxels found within the confines of the atlas maps.

**Supplementary Information Mx scripts**

**Brain mediation model**

**NGroups=1**

**Data Ninput=6 Nobservations=88**

**CMatrix**

**% voxelwise CMatrix**

**Label RTVS RTVB A FA MD DIS**

**Select RTVS RTVB A FA MD;**

**#define NumManifest 5**

**Begin Matrices;**

**S Symm 8 8**

**A Full 8 8**

**F Full 5 8**

**I Iden 8 8**

**End Matrices;**

**Matrix S**

**1**

**0 1**

**0 0 0**

**0 0 0 1**

**0 0 0 0 1**

**0 0 0 0 0 1**

**0 0 0 0 0 0 1**

**0 0 0 0 0 0 0 1**

**Specify S**

**1**

**0 2**

**0 0 0**

**0 0 0 3**

**0 0 0 0 4**

**0 0 0 0 0 0**

**0 0 0 0 0 0 0**

**0 0 0 0 0 0 0 0**

**Label Row S**

**RTVS RTVB A FA MD AGE BEH DTI**

**Label Col S**

**RTVS RTVB A FA MD AGE BEH DTI**

**Boundary -100 100 S 1 1 1**

**Boundary -100 100 S 1 2 2**

**Boundary -100 100 S 1 4 4**

**Boundary -100 100 S 1 5 5**

**Matrix A**

**0 0 0 0 0 0 1 0**

**0 0 0 0 0 0 1 0**

**0 0 0 0 0 1 0 0**

**0 0 0 0 0 0 0 1**

**0 0 0 0 0 0 0 1**

**0 0 0 0 0 0 0 0**

**0 0 0 0 0 0 0 0.5**

**0 0 0 0 0 0.5 0 0**

**Specify A**

**0 0 0 0 0 0 5 0**

**0 0 0 0 0 0 6 0**

**0 0 0 0 0 7 0 0**

**0 0 0 0 0 0 0 8**

**0 0 0 0 0 0 0 11**

**0 0 0 0 0 0 0 0**

**0 0 0 0 0 0 0 10**

**0 0 0 0 0 9 0 0**

**Label Row A**

**RTVS RTVB A FA MD AGE BEH DTI**

**Label Col A**

**RTVS RTVB A FA MD AGE BEH DTI**

**Boundary -100 100 A 1 1 7**

**Boundary -100 100 A 1 2 7**

**Boundary -100 100 A 1 3 6**

**Boundary -100 100 A 1 4 8**

**Boundary -100 100 A 1 5 8**

**Boundary -100 100 A 1 7 8**

**Boundary -100 100 A 1 8 6**

**Matrix F**

**1 0 0 0 0 0 0 0**

**0 1 0 0 0 0 0 0**

**0 0 1 0 0 0 0 0**

**0 0 0 1 0 0 0 0**

**0 0 0 0 1 0 0 0**

**Label Row F**

**RTVS RTVB A FA MD**

**Label Col F**

**RTVS RTVB A FA MD AGE BEH DTI**

**Covariance F & ((I - A)~ & S);**

**Option Multiple issat**

**End**

**drop 9**

**End**

**drop 10**

**End**

**End Group;**

**Cognitive mediation model**

**NGroups=1**

**Data Ninput=6 Nobservations=88**

**CMatrix**

**% voxelwise CMatrix**

**Label RTVS RTVB A FA MD DIS**

**Select RTVS RTVB A FA MD;**

**#define NumManifest 5**

**Begin Matrices;**

**S Symm 8 8**

**A Full 8 8**

**F Full 5 8**

**I Iden 8 8**

**End Matrices;**

**Matrix S**

**1**

**0 1**

**0 0 0**

**0 0 0 1**

**0 0 0 0 1**

**0 0 0 0 0 1**

**0 0 0 0 0 0 1**

**0 0 0 0 0 0 0 1**

**Specify S**

**1**

**0 2**

**0 0 0**

**0 0 0 3**

**0 0 0 0 4**

**0 0 0 0 0 0**

**0 0 0 0 0 0 0**

**0 0 0 0 0 0 0 0**

**Label Row S**

**RTVS RTVB A FA MD AGE BEH DTI**

**Label Col S**

**RTVS RTVB A FA MD AGE BEH DTI**

**Boundary -100 100 S 1 1 1**

**Boundary -100 100 S 1 2 2**

**Boundary -100 100 S 1 4 4**

**Boundary -100 100 S 1 5 5**

**Matrix A**

**0 0 0 0 0 0 1 0**

**0 0 0 0 0 0 1 0**

**0 0 0 0 0 1 0 0**

**0 0 0 0 0 0 0 1**

**0 0 0 0 0 0 0 1**

**0 0 0 0 0 0 0 0**

**0 0 0 0 0 0.5 0 0**

**0 0 0 0 0 0 0.5 0**

**Specify A**

**0 0 0 0 0 0 5 0**

**0 0 0 0 0 0 6 0**

**0 0 0 0 0 7 0 0**

**0 0 0 0 0 0 0 8**

**0 0 0 0 0 0 0 11**

**0 0 0 0 0 0 0 0**

**0 0 0 0 0 9 0 0**

**0 0 0 0 0 0 10 0**

**Label Row A**

**RTVS RTVB A FA MD AGE BEH DTI**

**Label Col A**

**RTVS RTVB A FA MD AGE BEH DTI**

**Boundary -100 100 A 1 1 7**

**Boundary -100 100 A 1 2 7**

**Boundary -100 100 A 1 3 6**

**Boundary -100 100 A 1 4 8**

**Boundary -100 100 A 1 5 8**

**Boundary -100 100 A 1 7 6**

**Boundary -100 100 A 1 8 7**

**Matrix F**

**1 0 0 0 0 0 0 0**

**0 1 0 0 0 0 0 0**

**0 0 1 0 0 0 0 0**

**0 0 0 1 0 0 0 0**

**0 0 0 0 1 0 0 0**

**Label Row F**

**RTVS RTVB A FA MD**

**Label Col F**

**RTVS RTVB A FA MD AGE BEH DTI**

**Covariance F & ((I - A)~ & S);**

**Option Multiple issat**

**End**

**drop 10**

**End**

**drop 9**

**End**

**End Group;**

**Independent factor model**

**NGroups=1**

**Data Ninput=6 Nobservations=88**

**CMatrix**

**% voxelwise CMatrix**

**Label RTVS RTVB A FA MD DIS**

**Select RTVS RTVB A FA MD ;**

**#define NumManifest 5**

**Begin Matrices;**

**S Symm 8 8**

**A Full 8 8**

**F Full 5 8**

**I Iden 8 8**

**End Matrices;**

**Matrix S**

**1**

**0 1**

**0 0 0**

**0 0 0 1**

**0 0 0 0 1**

**0 0 0 0 0 1**

**0 0 0 0 0 0 1**

**0 0 0 0 0 0 0 1**

**Specify S**

**1**

**0 2**

**0 0 0**

**0 0 0 3**

**0 0 0 0 4**

**0 0 0 0 0 0**

**0 0 0 0 0 0 0**

**0 0 0 0 0 0 0 0**

**Label Row S**

**RTVS RTVB A FA MD AGE BEH DTI**

**Label Col S**

**RTVS RTVB A FA MD AGE BEH DTI**

**Boundary -100 100 S 1 1 1**

**Boundary -100 100 S 1 2 2**

**Boundary -100 100 S 1 4 4**

**Boundary -100 100 S 1 5 5**

**Matrix A**

**0 0 0 0 0 0 1 0**

**0 0 0 0 0 0 1 0**

**0 0 0 0 0 1 0 0**

**0 0 0 0 0 0 0 1**

**0 0 0 0 0 0 0 1**

**0 0 0 0 0 0 0 0**

**0 0 0 0 0 0.5 0 0**

**0 0 0 0 0 0.5 0 0**

**Specify A**

**0 0 0 0 0 0 5 0**

**0 0 0 0 0 0 6 0**

**0 0 0 0 0 7 0 0**

**0 0 0 0 0 0 0 8**

**0 0 0 0 0 0 0 11**

**0 0 0 0 0 0 0 0**

**0 0 0 0 0 9 0 0**

**0 0 0 0 0 10 0 0**

**Label Row A**

**RTVS RTVB A FA MD AGE BEH DTI**

**Label Col A**

**RTVS RTVB A FA MD AGE BEH DTI**

**Boundary -100 100 A 1 1 7**

**Boundary -100 100 A 1 2 7**

**Boundary -100 100 A 1 3 6**

**Boundary -100 100 A 1 4 8**

**Boundary -100 100 A 1 5 8**

**Boundary -100 100 A 1 7 6**

**Boundary -100 100 A 1 8 6**

**Matrix F**

**1 0 0 0 0 0 0 0**

**0 1 0 0 0 0 0 0**

**0 0 1 0 0 0 0 0**

**0 0 0 1 0 0 0 0**

**0 0 0 0 1 0 0 0**

**Label Row F**

**RTVS RTVB A FA MD**

**Label Col F**

**RTVS RTVB A FA MD AGE BEH DTI**

**Covariance F & ((I - A)~ & S);**

**Option Multiple issat**

**End**

**drop 10**

**End**

**drop 9**

**End**

**End Group;**

**Common factor model**

**NGroups=1**

**Data Ninput=6 Nobservations=88**

**CMatrix**

**% voxelwise CMatrix**

**Label RTVS RTVB A FA MD DIS**

**Select RTVS RTVB A FA MD;**

**#define NumManifest 5**

**Begin Matrices;**

**S Symm 9 9**

**A Full 9 9**

**F Full 5 9**

**I Iden 9 9**

**End Matrices;**

**Matrix S**

**1**

**0 1**

**0 0 0**

**0 0 0 1**

**0 0 0 0 1**

**0 0 0 0 0 1**

**0 0 0 0 0 0 1**

**0 0 0 0 0 0 0 1**

**0 0 0 0 0 0 0 0 1**

**Specify S**

**1**

**0 2**

**0 0 0**

**0 0 0 3**

**0 0 0 0 4**

**0 0 0 0 0 0**

**0 0 0 0 0 0 0**

**0 0 0 0 0 0 0 0**

**0 0 0 0 0 0 0 0 0**

**Label Row S**

**RTVS RTVB A FA MD AGE BEH DTI com**

**Label Col S**

**RTVS RTVB A FA MD AGE BEH DTI com**

**Boundary -100 100 S 1 1 1**

**Boundary -100 100 S 1 2 2**

**Boundary -100 100 S 1 4 4**

**Boundary -100 100 S 1 5 5**

**Matrix A**

**0 0 0 0 0 0 1 0 0**

**0 0 0 0 0 0 1 0 0**

**0 0 0 0 0 1 0 0 0**

**0 0 0 0 0 0 0 1 0**

**0 0 0 0 0 0 0 1 0**

**0 0 0 0 0 0 0 0 0**

**0 0 0 0 0 0 0 0 0.5**

**0 0 0 0 0 0 0 0 0.5**

**0 0 0 0 0 0.5 0 0 0**

**Specify A**

**0 0 0 0 0 0 5 0 0**

**0 0 0 0 0 0 6 0 0**

**0 0 0 0 0 7 0 0 0**

**0 0 0 0 0 0 0 8 0**

**0 0 0 0 0 0 0 11 0**

**0 0 0 0 0 0 0 0 0**

**0 0 0 0 0 0 0 0 10**

**0 0 0 0 0 0 0 0 12**

**0 0 0 0 0 9 0 0 0**

**Label Row A**

**RTVS RTVB A FA MD AGE BEH DTI com**

**Label Col A**

**RTVS RTVB A FA MD AGE BEH DTI com**

**Boundary -100 100 A 1 1 7**

**Boundary -100 100 A 1 2 7**

**Boundary -100 100 A 1 3 6**

**Boundary -100 100 A 1 4 8**

**Boundary -100 100 A 1 5 8**

**Boundary -100 100 A 1 7 9**

**Boundary -100 100 A 1 8 9**

**Boundary -100 100 A 1 9 6**

**Matrix F**

**1 0 0 0 0 0 0 0 0**

**0 1 0 0 0 0 0 0 0**

**0 0 1 0 0 0 0 0 0**

**0 0 0 1 0 0 0 0 0**

**0 0 0 0 1 0 0 0 0**

**Label Row F**

**RTVS RTVB A FA MD**

**Label Col F**

**RTVS RTVB A FA MD AGE BEH DTI com**

**Covariance F & ((I - A)~ & S);**

**Option Multiple issat**

**End**

**drop 9**

**End**

**drop 12**

**End**

**drop 10**

**End**

**End Group;**

**Supplementary Information MATLAB scripts**

% Consider for your own analysis the new version of Mx which is coded in R and easy to run % through a loop <https://openmx.ssri.psu.edu/>

% Function:

% This function creates the parallel computing interface between classic Mx and MATLAB

% This routine reads A classic Mx script file and a voxel wise covariance matrix and

% estimates the SEM model. It gives a MATLAB output file.

% Input: Mx script MATLAB covar matrix

% Output: MATLAB File

% Hires: asciiread (NeuroElf https://neuroelf.net/), greppieBrMedWm_SRP and callmx_SRP

parpool

% Add NeuroElf to your path

addpath ('/YourPath/NeuroElf_v11_7251')

% Add the path were your data are

addpath ('/YourPath/')

% Load lower covar matrix

load ('FWF_RTVS_RTVB_A_FA_MD_DIS_masked.mat')

% Load Mx script

script=('/YourPath/BrMedWm.mx');

% Transfer mx script into MATLAB readable code

script = asciiread(script(:)');

l=length(kmatrix);

% Split the mx script in the header part (sciprt1) and the code part script (3)

script1 = script(1:100);

endscript=length(script);

script3 = script(100:endscript);

% Call MX in a parallel way

parfor i=1:l

% Read lower covar matrix numbers are string format

script2=kmatrix(i).KMATRIX;

% Insert covar matrix between header and script section of the Mx input file

scriptpar=[script1,script2,script3];

% Call mx function = input interface between MATLAB and Mx;

% Greppie function = output interface between Mx and MATLAB

vox(:,i)=greppieBrMedWm_SRP(callmx_SRP(scriptpar));

end

name={'RTVS' 'RTVB' 'FA' 'MD' 'DTI' 'BEH' 'fullmod_chi' 'dti_age_chi' 'dti_beh_chi' 'rmsea1' 'rmsea2' 'rmsea3' 'prob'};

file=('/YourPath/BrMedWmCo.mat');

save (file, 'vox', 'name');

delete(gcp)

% Consider for your own analysis the new version of Mx which is coded in R

% <https://openmx.ssri.psu.edu/>

% Function:

% This function transfers mxo string output (Mx output file) into MATLAB numbers

% It calls for MATLAB functions in NeuroElf https://neuroelf.net/

% Input: Mx output file (mxo)

% Output: MATLAB .mat file

% Hires: Regular expression

function [NII]=greppieBrMedWm_SRP(mxo)

addpath ('/YourPath/NeuroElf_v11_7251')

addpath ('/YourPath/')

% Finds all the "MATRIX A"

tabstart =strfind(mxo,'MATRIX A');

% Finds all the "MATRIX F"

tabend = strfind(mxo,'MATRIX F');

% Read the text between the Third 'MATRIX A' and the Third 'MATRIX F'

tab=mxo(tabstart(3):tabend(3)-1);

% Find position of 'DTI'

tab_stat=length(strfind(tab,'DTI'));

% Detect if model is corrupt

iscorrupt=((sum(strfind(mxo,'BLUE')))+(sum(strfind(mxo,'RED'))))>0;

try

% If model is corrupt all output is zero

if iscorrupt>0

NII(1:13)=0;

% If model is correct and the postion of dti == 3 than extract string and transfer into % number

elseif tab_stat==3

RegExpression1='\s*(RTVS)(\s+([0-9\.\-\+\e]+)){0}\s*';

RegExpression2='\s*(RTVS)(\s+([0-9\.\-\+\e]+)){1}\s*';

startpos=regexp(tab,RegExpression1,'end');

endpos=regexp(tab,RegExpression2,'end');

NII(1)=str2double(tab(startpos(3):endpos(2)));

RegExpression1='\s*(RTVB)(\s+([0-9\.\-\+\e]+)){0}\s*';

RegExpression2='\s*(RTVB)(\s+([0-9\.\-\+\e]+)){1}\s*';

startpos=regexp(tab,RegExpression1,'end');

endpos=regexp(tab,RegExpression2,'end');

NII(2)=str2double(tab(startpos(3):endpos(2)));

RegExpression1='\s*(FA)(\s+([0-9\.\-\+\e]+)){1}\s*';

RegExpression2='\s*(FA)(\s+([0-9\.\-\+\e]+)){2}\s*';

startpos=regexp(tab,RegExpression1,'end');

endpos=regexp(tab,RegExpression2,'end');

NII(3)=str2double(tab(startpos(2):endpos(2)));

RegExpression1='\s*(MD)(\s+([0-9\.\-\+\e]+)){1}\s*';

RegExpression2='\s*(MD)(\s+([0-9\.\-\+\e]+)){2}\s*';

startpos=regexp(tab,RegExpression1,'end');

endpos=regexp(tab,RegExpression2,'end');

NII(4)=str2double(tab(startpos(2):endpos(2)));

RegExpression1='\s*(DTI)(\s+([0-9\.\-\+\e]+)){5}\s*';

RegExpression2='\s*(DTI)(\s+([0-9\.\-\+\e]+)){6}\s*';

startpos=regexp(tab,RegExpression1,'end');

endpos=regexp(tab,RegExpression2,'end');

NII(5)=str2double(tab(startpos(1):endpos(1)));

RegExpression1='\s*(BEH)(\s+([0-9\.\-\+\e]+)){1}\s*';

RegExpression2='\s*(BEH)(\s+([0-9\.\-\+\e]+)){2}\s*';

startpos=regexp(tab,RegExpression1,'end');

endpos=regexp(tab,RegExpression2,'end');

NII(6)=str2double(tab(startpos(2):endpos(2)));

mxstart=strfind(mxo,'Chi-squared fit of model >>>>>>>');

RegExpression1='(\s+([0-9\.\-\+\e]+))\s*';

NII(7) = str2double(regexp(mxo(mxstart(1)+32:mxstart(1)+50),RegExpression1,'match'));

NII(8) = str2double(regexp(mxo(mxstart(2)+32:mxstart(2)+50),RegExpression1,'match'));

NII(9) = str2double(regexp(mxo(mxstart(3)+32:mxstart(3)+50),RegExpression1,'match'));

mxstart=strfind(mxo,'RMSEA >>>>>>>>>>>>>>>>>>>>>>>>>>');

RegExpression1='(\s+([0-9\.\-\+\e]+))\s*';

NII(10) = str2double(regexp(mxo(mxstart(1)+32:mxstart(1)+50),RegExpression1,'match'));

NII(11) = str2double(regexp(mxo(mxstart(2)+32:mxstart(2)+50),RegExpression1,'match'));

NII(12) = str2double(regexp(mxo(mxstart(3)+32:mxstart(3)+50),RegExpression1,'match'));

mxstart=strfind(mxo,'Probability >>>>>>>>>>>>>>>>>>>>');

RegExpression1='(\s+([0-9\.\-\+\e]+))\s*';

Prob = str2double(regexp(mxo(mxstart(1)+32:mxstart(1)+50),RegExpression1,'match'));

NII(13) = Prob(1);

else

NII(1:13)=0;

end

catch

NII(1:13)=0;

end

clear tab

% Consider for your own analysis the new version of Mx which is coded in R

% <https://openmx.ssri.psu.edu/>

% This function creates the interface between mx and MATLAB and collects the mxo (mx output) files

function [mxo] = callmx_SRP(scriptpar)

addpath ('/YourPath/NeuroElf_v11_7251')

% Generate temp file

tfile = strrep([tempdir filesep strrep(tempname, filesep, '_') sprintf('%06.0f', 1e6*rand(1,1))], ...

[filesep filesep], filesep);

asciiwrite([tfile '.mx'], scriptpar);

% Call mx

[~,mxo] = system(['/bin/cat ' tfile '.mx | mx_init']);

delete([tfile '.mx']);

% Consider for your own voxel wise analysis the new version of Mx which is coded in R and % therefore much easier to handle

% <https://openmx.ssri.psu.edu/>

% This function reads response times and DTI data masked for FA>0.2 and estimates covariance % matrices for the masked data that are readable in classic Mx

parpool

% Add path were neuroelf is found

addpath ('/YourPath/NeuroElf_v11_7251')

% Add path where your data are

addpath (‘/YourPath/’)

% Load response data

load RT.mat

% Load DTI data

load Subjects_DTI.mat

% Working memory response times of test and retest run are averaged

RT_VS=(rtSternVS_a+rtSternVS_b)./2;

RT_VB=(rtSternVB_a+rtSternVB_b)./2;

% This uses a mask with FA larger 0.2

load /YourPath/mask02.mat

vox_selection=find(mask==1);

lvs=length(vox_selection);

% Reserve covariance matrix of size 6*6*length mask

CoMT=ones(6,6,lvs);

% This is to extract data within mask

total=Proband;

ziel=(Subjects_DTI);

[c] = intersect(ziel, total);

di=ismember(ziel,c);

indi=find(di==0);

ziel(indi)=[];

for da =1:length(ziel)

temp=strcmp(ziel(da),total);

selection(da)=find(temp==1);

end

% Transfer 3D imaging data into 1D

fa=xff('/YourPath/all_FA_neu.nii');

s=size(fa.VoxelData);

row=s(1)*s(2)*s(3);

col=s(4);

quattro_fa=fa.VoxelData(:,:,:,1:88);

duofa=(reshape(quattro_fa,row,col));

duofa(:,indi)=[];

duofa=(duofa(vox_selection,:))';

fa.ClearObject

md=xff('YourPath/all_MD_neu.nii');

quattro_md=md.VoxelData(:,:,:,1:88);

duomd=(reshape(quattro_md,row,col));

duomd(:,indi)=[];

duomd=(duomd(vox_selection,:))';

md.ClearObject

% Sort RT times and age and standardize RT times and age from 0 to 10

RTVS = (RT_VS(selection));

RTVS = (RTVS-min(RTVS))./(max(RTVS)-min(RTVS)).*10;

RTVB = (RT_VB(selection));

RTVB = (RTVB-min(RTVB))./(max(RTVB)-min(RTVB)).*10;

DIS = (((RT_Distr_VB(selection))+(RT_Distr_VS(selection)))./2);

DIS = (DIS-min(DIS))./(max(DIS)-min(DIS)).*10;

A = (Alter(selection));

A = (A-min(A))./(max(A)-min(A)).*10;

% Create covariance matrix for Mx using parallel computing

parfor i = 1:lvs

duofatemp = duofa(:,i);

duofatemp = -1.*(duofatemp-min(duofatemp))./(max(duofatemp)-min(duofatemp)).*10;

duomdtemp = duomd(:,i);

if (min(duomdtemp))>0

duomdtemp = (duomdtemp-min(duomdtemp))./(max(duomdtemp)-min(duomdtemp)).*10;

else

duomdtemp =(duomdtemp+abs(min(duomdtemp)))./(max(duomdtemp)+abs(min(duomdtemp))).*10;

end

CoMT(:,:,i)=cov([RTVS RTVB A duofatemp duomdtemp DIS]);

kmatrix(i).KMATRIX='';

end

% Transfer covariance matrix into Mx readable format

parfor i = 1:lvs

CoM=CoMT(:,:,i);

for x=1:6

if CoM(x,1)<0,

emptyspace=' ';

else

emptyspace=' ';

end

kmatrix(i).KMATRIX=[kmatrix(i).KMATRIX,emptyspace,num2str(CoM(x,1:x),'%15.4f')];

kmatrix(i).KMATRIX=[kmatrix(i).KMATRIX,sprintf('\n')];

end

end

save (['YourPath/FWF_RTVS_RTVB_A_FA_MD_DIS_masked.mat'], 'kmatrix');

delete(gcp)
